# Medical Care for Tuberculosis-HIV-Coinfected Patients in Russia with Respect to a Changeable Patients’ Structure

**DOI:** 10.3390/tropicalmed7060086

**Published:** 2022-05-31

**Authors:** Olga P. Frolova, Olga V. Butylchenko, Patimat G. Gadzhieva, Margarita Yu. Timofeeva, Valeria A. Basangova, Vladislava O. Petrova, Inna A. Fadeeva, Maria I. Kashutina, Nadezhda N. Zabroda, Artem A. Basov, Elena V. Belova, Yury V. Zhernov, Oleg V. Mitrokhin, Inga I. Enilenis, Lyudmila P. Severova

**Affiliations:** 1 M.I. Perelman Department of Phthisiopulmonology and Thoracic Surgery, I.M. Sechenov First Moscow State Medical University (Sechenov University), 119991 Moscow, Russia; opfrolova@yandex.ru (O.P.F.); olga16.53@list.ru (O.V.B.); schuldich9@yandex.ru (P.G.G.); lera.basangova@yandex.ru (V.A.B.); enilinga@yandex.ru (I.I.E.); 2 Department of Phthisiology, Pirogov Russian National Research Medical University, 117997 Moscow, Russia; 3 Department of Medical Law, I.M. Sechenov First Moscow State Medical University (Sechenov University), 119991 Moscow, Russia; timofeevainfo@mail.ru; 4 Department of General Hygiene, F. Erismann Institute of Public Health, I.M. Sechenov First Moscow State Medical University (Sechenov University), 119435 Moscow, Russia; vlada.petrova01@mail.ru (V.O.P.); zabroda_n_n@staff.sechenov.ru (N.N.Z.); basov_a_a@staff.sechenov.ru (A.A.B.); ms.ekochina@mail.ru (E.V.B.); mov1163@yandex.ru (O.V.M.); 5 Department of English Language, Institute of World Economy, Diplomatic Academy of the Russian Foreign Ministry, 119034 Moscow, Russia; innaf576@mail.ru; 6 Loginov Moscow Clinical Scientific and Practical Center, 111123 Moscow, Russia; kashutina.maria@gmail.com; 7 National Research Center for Therapy and Preventive Medicine, 101990 Moscow, Russia; 8 Department of Chemistry, Lomonosov Moscow State University, 119991 Moscow, Russia

**Keywords:** HIV, AIDS, tuberculosis, drug resistance, social maladaptation

## Abstract

To date, tuberculosis (TB) remains the primary cause of mortality in human immunodeficiency virus (HIV) patients in Russia. Since the beginning of 2000, a sharp change in the HIV patients’ structure, to the main known risk factors for HIV infection has taken place in Russia. The transmission of HIV through injectable drug use has begun to decline significantly, giving way to the prevalence of sexual HIV transmission today. These changes may require adjustments to organizational approaches to anti-TB care and the treatment of HIV-positive patients. Our study is aimed at identifying changes in TB-HIV coinfection patients’ structures in 2019 compared to 2000. Based on the results obtained, our goal was to point out the parameters that need to be taken into account when developing approaches to improve the organization of TB control care for people with HIV infection. We have carried out a cross-sectional, retrospective, epidemiological study using government TB registry data from four regions in two federal districts of Russia in 2019. The case histories of 2265 patients from two regions with high HIV prevalence, which are part of the Siberian Federal District of Russia, and 89 patient histories from two regions of low HIV prevalence, which are part of the Central Federal District of Russia, were analyzed. We found that parenteral transmission (69.4%) remains the primary route of HIV transmission among the TB-HIV coinfected. The unemployed of working age without disability account for 80.2% of all coinfected people, while the formerly incarcerated account for 53.7% and the homeless account for 4.1%. Those with primary multidrug-resistant TB (MDR-TB) comprise 56.2% of HIV-TB patients. When comparing the incidence of coinfection with HIV among TB patients, statistically significant differences were obtained. Thus, the chances of coinfection increased by 4.33 times among people with active TB (95% CI: 2.31; 8.12), by 2.97 times among people with MDR-TB (95% CI: 1.66; 5.32), by 5.2 times in people with advanced processes in the lungs, including destruction, (95% CI: 2.78; 9.7), as well as by 10.3 times in the case of death within the first year after the TB diagnosis (95% CI: 2.99; 35.5). The absence of data for the presence of TB during preventive examination was accompanied by a decrease in the chances of detecting coinfection (OR 0.36; 95% CI: 0.2; 0.64). We have identified the probable causes of the high incidence of TB among HIV-infected: HIV-patient social maladaptation usually results in delayed medical care, leading to TB treatment regimen violations. Furthermore, self-administration of drugs triggers MDR-TB within this group. Healthcare providers should clearly explain to patients the critical importance of immediately seeking medical care when initial TB symptoms appear.

## 1. Introduction

The human immunodeficiency virus infection and acquired immunodeficiency syndrome (HIV/AIDS) epidemic is one of humanity’s most acute problems. In 2020, according to UNAIDS, the number of people living with HIV was 37.7 million. There were 1.5 million people newly infected with HIV in 2020. In 2020 there were 680,000 AIDS-related deaths [1].

In macro-regions of the world, including Russia, the HIV/AIDS epidemic is genetically heterogeneous due to the many types of HIV and the circulating recombinant forms (CRF) [2]. In Russia, according to personalized records as of 2019, the total number of registered cases of HIV infection reached 1.4 million, of which 355 thousand HIV-infected people died within the period of HIV surveillance [3]. Eighty-nine thousand people were infected with HIV and 618 people died in Russia in 2000 [4].

It should be noted that the current unfortunate situation concerning the HIV/AIDS epidemic in Russia is the result of the historical peculiarities of the country’s development. From the late 1980s until the first half of the 1990s, the HIV/AIDS epidemic in Russia and neighboring countries was sporadic. The situation changed dramatically in the second half of the 1990s due to an avalanche-like spread of infection among injecting drug users (IDUs) in Russia and neighboring countries with a predominance of HIV-1 subtype A1 [5,6]. Another feature of the HIV/AIDS epidemic in Russia is its frequent concomitant course with tuberculosis (TB). In Russia, TB is the leading cause of death in HIV-infected patients [7,8,9].

Thus, the problem of TB in HIV patients continues to be of vital relevance in Russia. In addition, the WHO noted the importance of solving this crisis in its 2020 Global TB Report [10]. The Russian Federal AIDS Center data reveal a change in the HIV-positive patient structure, particularly concerning the age and gender of groups and the modes of transmission [7,8]. These changes may require adjustments to organizational approaches for anti-TB care and the treatment of HIV patients.

In the period from 2000 to 2019, the number of young HIV and HIV-TB patients aged 15 to 29 years has significantly decreased in Russia. The number of men with HIV and HIV-TB has decreased overall. However, the opposite trend was noted among the female population in Russia. The number of intravenous drug users among patients with HIV and HIV-TB has greatly decreased. The number of HIV-infected people and risk factors for infection are changing.

The current TB screening system is not effective for patients with HIV. The current approach to prevention needs to be adjusted since it does not consider the specifics of TB development against the background of immunodeficiency. MDR-TB is more common in patients with HIV and is most likely caused by low treatment compliance due to the social isolation that is more common in the main group of patients.

To avoid the development of multidrug-resistant TB (MDR-TB) and the immune reconstitution inflammatory syndrome (IRIS) when working with HIV-infected people from among socially isolated citizens, attention should be paid to the need to use additional methods of information and motivation.

Consequently, our study aims to ascertain changes in TB-HIV coinfected patients’ structure in 2019 compared to 2000, highlighting points that need to be considered to enhance the organization of anti-TB care for HIV patients.

## 2. Materials and Methods

This is a cross-sectional, retrospective, epidemiological study using government TB registry data from four regions in two federal districts of Russia in 2019. The case histories of 2265 patients from two regions with high HIV prevalence, which are part of the Siberian Federal District of Russia, and 89 patient histories from two regions of low HIV prevalence, which are part of the Central Federal District of Russia, were analyzed.

The study was conducted in accordance with the Declaration of Helsinki, and approved by the Local Ethic Committee of the I.M. Sechenov First Moscow State Medical University (Sechenov University) (protocol № 08-22, 20 April 2022). Informed consent was obtained from all subjects involved in the study.

The collected data of all 2354 patients was entered into individual cards (questionnaires), consisting of 28 blocks (Table S1) [11]. Thus, socio-demographic characteristics were analyzed, including stays in places of detention as well as the ways of HIV transmission, the dates of detection of both infections, the forms of TB, the frequency of bacterial excretion, drug resistance, and mortality. Data on HIV-TB patients from four regions in two Russian federal districts were compared to official published data from the Russian Federal AIDS Center on the total number of HIV patients in Russia [7,8] and data on TB patients in Russia without HIV published in TB reports for 2019 and 2000 [9].

In addition, with 62 patients who filled out questionnaires, confidential interviews were conducted and analyzed to assess compliance with medical prescriptions. We checked the observations of patients who were given anti-TB drugs free of charge in 2019.

To compare relative data between years, we used descriptive statistics. To compare the nominal variables, a Pearson’s chi-square test was used with the determination of the odds ratio (OR) and 95% confidence interval (95% CI), as well as the assessment of the strength of the relationship between the features using Cramer’s V-value (the interpretation of the Cramer’s V-values of which was carried out in accordance with the data: the strength of the relationship is insignificant if the value is <0.1, weak: 0.1–<0.2, average: 0.2–<0.4, relatively strong: 0.4–<0.6, strong: 0.6–<0.8, very strong: 0.8–1.0).

## 3. Results

**Figure 1:**
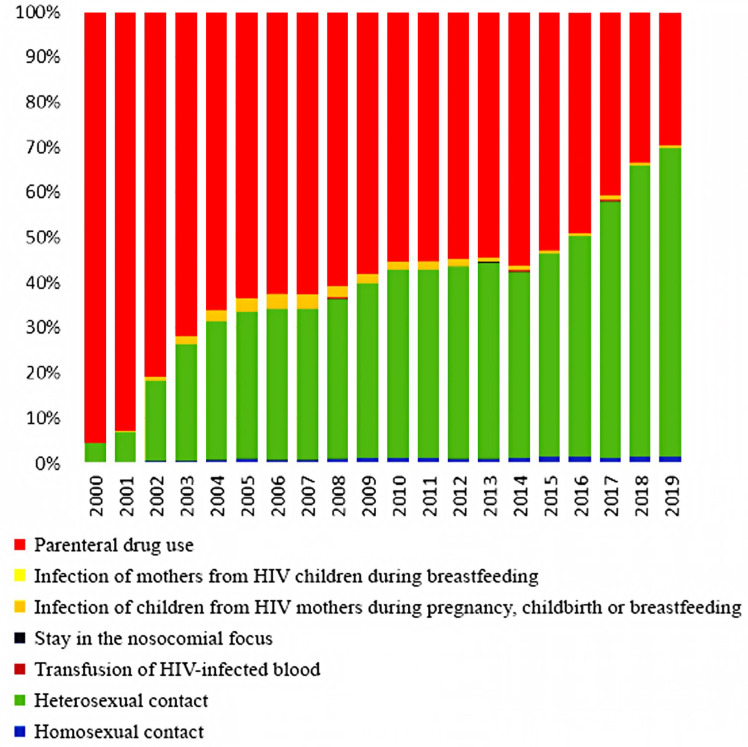
The HIV patients’ structure by the main known infection factors in Russia from 2000 to 2019 (according to the Russian Federal AIDS Center).

The comparison of data from our cross-sectional, retrospective, epidemiological study of HIV-TB patients in 2019 with data from registers of 2000 is due to the fact that since the beginning of 2000, a sharp change in the HIV patients’ structure according to the main known risk factors for HIV infection has taken place in Russia. The transmission of HIV through injectable drugs has begun to decline significantly, giving way to the prevalence of sexual HIV transmission. The presented data from official statistics clearly show these changes in contingents (Figure 1). These changes may require adjustments to organizational approaches for the anti-TB care and treatment of HIV-positive patients.

**Figure 2:**
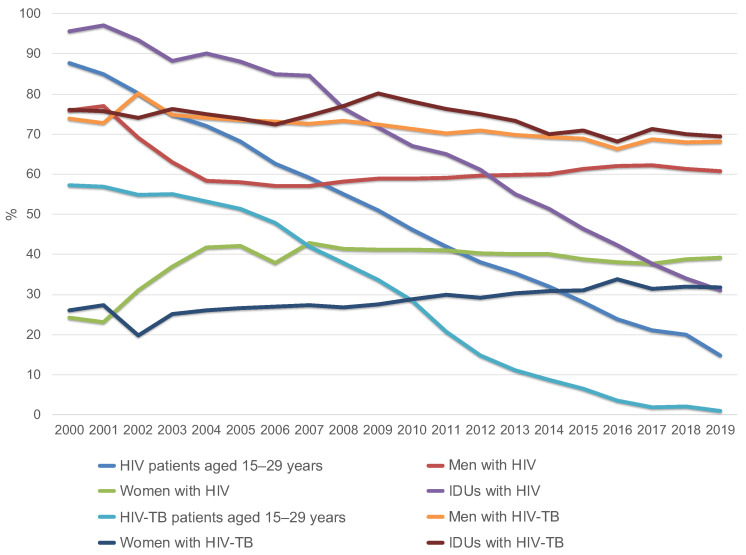
Changes in the structure of HIV patients and HIV-TB patients by age (group of 15–29 year old’s), gender and injecting drug use in Russia from 2000 to 2019 (according to the Russian Federal AIDS Center).

Dynamic monitoring of the entire group of HIV patients, both infected and uninfected with TB, conducted by the Russian Federal AIDS Center, showed that the proportion of HIV patients aged 15–29 years was 87.6% in 2000 [8], which decreased to 14.7% in 2019 [1]. In 2000, the proportion of HIV-TB patients aged 15–29 years was 57.3% (no mean age available), but by 2019 it had dropped to 1.0% (mean age = 44.3 years) (Figure 2, Table 1) [12,13].

Among all newly diagnosed HIV patients, both infected and uninfected with TB, men accounted for 60.8% of cases in 2019 [14], and among HIV-TB patients, 68.2%, which corresponds to the total number of TB patients in the general Russian population [9,15].

Among the entire group of HIV patients in 2019 and 2020, the main transmission route was sexual [7,8]. The proportion of drug users decreased to 33% in 2019 and 31% in 2020 [8,14] compared to 95.6% in 2000 [14,16]. At the same time, the proportion of drug users among patients with HIV-TB coinfection practically does not decrease. This group makes up the designated (reduced compared to 2000) percentage of HIV-infected people with intravenous drug use. In 2000, 76% of HIV-TB patients received intravenous injections; in 2019, the figure was 69.4% [13].

The social characteristics of HIV-infected patients diagnosed with TB in 2019 were analyzed: the unemployed of working age without disability constitute 80.2% of cases; those with disabilities account for 13.3%; the homeless make up 4.1%; and the previously imprisoned 53.7%, of which 57.1% were in prison for more than three years. Between 2000 and 2019, rates did not change significantly [12,13] (Table 2).

Some variables create difficulties with TB treatment in HIV-positive patients. As of 2019, a significant proportion of HIV-TB patients had widespread TB infection with destruction and dissemination (78.6%) and primary drug resistance (PDR) to anti-TB drugs. This problem was not relevant for patients without HIV (Table 3) [9].

When comparing the incidence of coinfection with HIV among TB patients, statistically significant differences were obtained. Thus, the chances of detecting coinfection increased by 4.33 times among people with active TB (95% CI: 2.31; 8.12), by 2.97 times among people with MDR-TB (95% CI: 1.66; 5.32), by 5.2 times in people with advanced processes in the lungs, including destruction, (95% CI: 2.78; 9.7), as well as by 10.3 times in the case of death within the first year after the TB diagnosis (95% CI: 2.99; 35.5). The absence of data for the presence of TB during preventive examination was accompanied by a decrease in chances of detecting coinfection (OR 0.36; 95% CI: 0.2; 0.64). An average relationship was noted between all compared parameters, in accordance with Cramer’s V-value.

Data from the cross-sectional, retrospective, epidemiological study showed that in 2019, *Mycobacterium tuberculosis* was found in the biological fluids of 79.6% of HIV-TB patients. In this group of patients with primary MDR-TB, simultaneous resistance to two main anti-TB drugs, rifampicin and isoniazid, was detected in 41.0% of cases in 2010 [17], while its overall share was growing to 56.2% of patients in 2019. In TB patients without HIV infection, MDR-TB was registered in 30.1% of cases, and bacterial excretion was found in 42.8% in 2019 [9]. In addition, 68.9% of HIV-TB patients had hepatitis C, of which 4.2% were concurrently infected with hepatitis B.

In Russia, TB in HIV patients was identified more often when patients were presented to hospitals because of TB symptomatic complaints (63.4% of our patients) and not during preventive examinations. On the other hand, among the entire group of TB patients without HIV, TB was more often detected during medical screening and preventive counseling in 62% of patients in 2019 [9]. When patients presented to district clinics with complaints related to TB, 12.1% of cases were HIV positive for the first time.

Among the entire group of all HIV-TB patients in our study (*n* = 2354), the duration of the HIV infection course from the moment of diagnosis to TB detection was more than ten years in 30.6% of cases, from 10 to 5 years in 28.8%, from five years to a year in 32.9%, and less than a year in 7.7%. Just over 13 percent had a relapse of TB, from which they suffered for the first time even before being infected with HIV, and 7.2% had a relapse of TB after HIV detection. When TB was diagnosed in patients with HIV infection, 9.9% of patients had a CD4 lymphocyte counts to greater than 500 cells/mm^3^, 24.8%—from 500 to 200, 48.3%—from 200 to 150, and 17.0%—less than 50.

Patients registered for HIV infection seek medical assistance in the Russian Federal AIDS Center when complaints appear, and those who are not registered seek treatment at their local clinics. Thirteen percent of HIV cases were detected during hospitalization in general somatic or infectious hospitals due to the severity of present medical conditions. Twenty-four percent of patients died within a year following the detection of TB, and 2.5% of TB cases in 2019 caused death in the absence of HIV infection within a year after the detection of TB [9].

During our study, at the stage of confidential interviews with 62 HIV patients who received anti-TB drugs for chemoprophylaxis, 32.3% admitted that they either did not take them at all, or started taking them, but stopped after one to four weeks, or took them irregularly.

Furthermore, our analysis of lethal outcomes during the year after TB identification in HIV patients revealed that coinfection (ICD-10 code: B20.0) was the direct cause of death in only 36.2% of cases. Accidents occurred in 3.2% of cases. In the remaining four percent multiple infections (ICD-10 code: B20.7) and multiple organ failure were the causes of death.

## 4. Discussion

The comparison of the whole group of HIV patients in 2019 in the entire Russian Federation and those with TB in the four considered regions of the Russian Federation in 2019 reveals corresponding changes in the demographics and routes of infection in the study groups (Table 1).

In the group of newly diagnosed HIV-infected TB patients, there is more bacterial shedding and multidrug resistance than in the group without HIV infection. To a greater extent, the deterioration of the clinical picture and subsequent statistics explain the inherent HIV-induced depletion of CD4+ T-lymphocytes as the main target of the virus, as well as macrophages. Immunodeficiency greatly accelerates the course of any inflammatory process, and especially that caused by *Mycobacterium tuberculosis*, since TB is also able to exert its own influence on HIV, increasing the level of replication and genetic diversity of the virus. This “collaboration” provides mutual benefits to the pathogens [18,19].

TB in HIV becomes more aggressive. Thus, among patients with coinfection, a widespread process or destruction in the lungs occurred in 78.6% of cases, while in patients with TB without HIV, it was only in 41.6% [9]. However, an earlier study on the causes of death in a group of HIV-TB patients [20] in five regions of the Russian Federation revealed that a lethal outcome was not associated only with TB in half of the cases. The most frequent causes of death include several secondary infections with wasting syndrome (ICD-10 codes: B20.7 and B22.2) in 19.7% of cases; other secondary infections—in 27.1%; cirrhosis of the liver developed as a result of hepatitis C or alcoholism—in 18.7%; drug overdose—in 12.3%; accidents—in 4.6% [20].

The social side of the problem should also be considered. People from the main HIV-TB risk group are characterized by social exclusion to varying degrees [21]. This feature explains the latter’s seeking medical help even in acute conditions, which excludes early diagnosis of an existing disease and reduces the likelihood of successful treatment. It also suggests a lack of understanding of possible risks and low motivation in treatment, which leads to a violation of the rules for taking medications in case of their free distribution and drug resistance because of such behavior (PDR-TB) [22,23,24].

The situation with the use of ART across the country does not look better. According to all-Russian statistics, 38.0% of patients with HIV infection refused to take antiretroviral drugs before the onset of TB. Eighteen percent of patients take them irregularly. In this scenario, preventing MDR-TB and improving the efficacy of ART is possible only with the help of constant monitoring of patient compliance with a drug regimen by a healthcare professional, which is not possible in an outpatient setting.

A complete cessation of the free distribution of ART and anti-TB drugs to such citizens is also not an option to solve the problem. Such an approach will not contribute to the formation of a more responsible attitude towards the treatment regimen since the level of well-being of such people often makes it difficult to independently purchase drugs, which may even worsen the situation in general. Thus, the availability of ART reduces the risk of TB in HIV patients by 58–80% [25]. Taking any anti-TB drug reduces the risk of developing active TB by 32% in people living with HIV in general, and by 62% in people with a positive tuberculin skin test [26].

However, it is necessary to adjust the order of distribution of anti-TB drugs concerning chemoprophylaxis [27]. Low adherence to the treatment regimen is the first thing that MDR is explained by. As a result, we get an undertreated patient who is able to pass an altered form of TB to others, for which standard treatment regimens are powerless. Currently, among HIV-TB patients, primary MDR-TB in anti-TB drugs occurs in 56.2% of cases, while in people without HIV infection it is only 30.1% [11]. How to improve the situation without resorting to a complete restriction on the availability of drugs is still an open question. Perhaps such patients should contact a mental health professional. Mandatory work of the patient with such a specialist can increase adherence to the prescribed pharmacotherapy.

The currently existing system of prevention, including, first of all, preventive examinations and routine X-ray diagnostics, carried out at a certain time interval, is effective in patients with slowly developing TB without HIV (detection in 62.0%, at an early stage without lung destruction, 58.4%) [11], but for HIV-TB patients it is almost ineffective since it does not take into account the more rapid progression of TB in immunodeficient conditions [19,20] (in 78.6% of cases, there is already widespread process with destruction and dissemination). It is noted that changes in the X-ray pattern in miliary TB appear later than their clinical manifestations in immunodeficiency conditions [28]. Thus, the main clinical manifestations appear first, and changes in radiological diagnostics can be seen, and the detection of bacteria in body fluids can be made only after about 15 days [29,30]. Therefore, more often, TB-HIV is detected after the appearance of complaints.

It is worth adding to the above that it is usually difficult to involve HIV-infected people from high-risk groups of TB infection in regular preventive examinations. As already noted, in most cases, TB occurred in socially maladapted HIV-infected people who were not registered with the Russian Federal AIDS Center and, as a result, did not undergo medical examination.

In the context of the traditional statistical assessment of TB care in the Russian Federation, the first detection of TB upon contacting a polyclinic with a complaint is considered a negative criterion, which indicates an imminent future deterioration in statistics with this approach as the proportion of HIV infection among TB patients increases. In 2009 it was 6.5%, and in 2019 it was already 24.7% [9].

Late diagnosis of TB in HIV patients increases the risk of one of the most dangerous complications of ART in TB. One of two forms of IRIS should be considered after starting ART and the subsequent rapid recovery of immune cells. Paradoxical TB can occur with a high degree of probability within three months from the start of ART in the case of previously diagnosed TB in the patient if there is a response to anti-TB treatment. Unmasking will occur if TB is not detected before the start of ART (ART-associated IRIS) [31,32,33,34].

A promising direction of research in the field of early diagnosis of TB in HIV patients is the identification and use of a group of markers, such as HLA-DR [35], as well as the further development of a system of genotypic tests based on the amplification of *Mycobacterium tuberculosis* nucleic acids [36]. These inventions will increase the likelihood of detecting TB in HIV at an early stage of development and drug sensitivity of a particular pathogen.

## 5. Conclusions

This study shows the urgent need to adjust existing approaches to the treatment and prevention of HIV-TB, considering the characteristics inherent in the main risk group. Patients should be sufficiently motivated to seek medical help immediately after the first signs of TB appear. The development of a system of information and psychological support for socially maladjusted patients when prescribing treatment can help reduce the risk of MDR-TB with uncontrolled intake of anti-TB drugs prescribed as chemoprophylaxis.

However, the way the virus spreads is changing, and the main means of transmission is shifting towards the sexual transmission of HIV. The need to develop new methods of minimally invasive, affordable prevention, including local action, comes to the fore [37]. Thus, microbicides, acting directly or indirectly, can restrain the release of an infectious agent into the human body, thereby preventing sexual HIV transmission and a number of other diseases [37,38].

## Figures and Tables

**Table 1:** Dynamics of change among all HIV-positive patients and among HIV-TB patients sorted by age (group of 15–29 year old’s), gender, and injecting drug use in Russia in 2019 compared to 2000 (* based on Russian Federal AIDS Center data; ** based on a cross-sectional retrospective epidemiological study).

	2000		2019	
	HIV-Patients (*n* = 89,808) *	HIV-TB Patients	HIV-Patients (*n* = 1,078,513) *	HIV-TB Patients (*n* = 2354) **
Patients aged 15–29 years	87.6%	57.3%	14.7%	1.0%
	(*n* = 78,672)		(*n* = 158,541)	(*n* = 24)
Men	75.9%	73.9%	60.8%	68.2%
	(*n* = 68,165)		(*n* = 655,735)	(*n* = 1606)
Women	24.1%	26.1%	39.2%	31.8%
	(*n* = 21,644)		(*n* = 422,777)	(*n* = 749)
IDUs	95.6%	76%	31.1%	69.4%
	(*n* = 85,856)		(*n* = 335,417)	(*n* = 1634)

**Table 2:** Social status of HIV-TB patients in Russia in 2019 compared to 2000 (* based on cross-sectional retrospective epidemiological study).

Social Characteristics	2000	2019 (*n* = 2354) *	Significance Level (*p*)
Unemployed of working age without disability	77.3%	80.2% (*n* = 1888)	0.61
Homeless people	4.3%	4.1% (*n* = 97)	0.73
Previously held in penitentiaries/formerly incarcerated	52.2%	53.7% (*n* = 1264)	0.78

**Table 3:** Characteristics of HIV-TB patients in comparison to patients with only TB infection in 2019 (TB—tuberculosis; MDR-TB—multidrug-resistant TB; OR—odds ratio; 95% CI—95% confidence interval).

	TB Patients (*n* = 126,737)	HIV-TB Patients (*n* = 2265)	Significance Level (*p*)	Cramer’s V-Value	OR; 95% CI
People with active TB	48.2%	79.6%	<0.001	0.333	4.33
	(*n* = 61,088)	(*n* = 1874)			(2.31; 8.12)
People with MDR-TB	30.1%	56.2%	<0.001	0.263	2.97
	(*n* = 38,148)	(*n* = 1323)			(1.66; 5.32)
People with advanced processes in lungs or destruction	41.6%	78.6%	<0.001	0.378	5.2
	(*n* = 52,722)	(*n* = 1851)			(2.78; 9.7)
Diagnostic rate of TB during the annual preventive examination	62.0%	36.6%	<0.001	0.250	0.36
	(*n* = 78,577)	(*n* = 862)			(0.2; 0.64)
Death rate within the first year after TB diagnosis	2.5%	24.4%	<0.001	0.307	10.3
	(*n* = 3169)	(*n* = 575)			(2.99; 35.5)

## Data Availability

The data presented in this study are available on request from the corresponding author.

## Supplementary Materials

The following supporting information can be downloaded at: https://www.mdpi.com/article/10.3390/tropicalmed7060086/s1, Table S1: Chemoprophylaxis Questionnaire for TB Patients with HIV Infection.
