# Supplementary material for: Medical Care for Tuberculosis-HIV-Coinfected Patients in Russia with Respect to a Changeable Patients’ Structure
Source: Trop Med Infect Dis. 2022 May 31;7(6):86. doi: 10.3390/tropicalmed7060086 (PMC9228798; doi:10.3390/tropicalmed7060086)
Supplement: Supplementary file 1 [file tropicalmed-07-00086-s001.zip › tropicalmed-1694663-supplementary.pdf]

Tropical Medicine and Infectious Disease

MDPI

Supplementary Materials:

Table S1. Questionnaire on chemoprophylaxis for patients with tuberculosis-HIV-coinfection

1	Number of the medical card of an outpatient patient in the Central district clinic of ________________(region)	
2	Sex/gender	
3	Date of birth/age	
4	Place of residence (city/village)	
5	Occupation, employment status, disability	
6	Date of registration of HIV infection	
7	Circumstances under which HIV infection was detected (complaints, active detection, etc.)	
8	Period of time in which HIV infection could have occurred	
9	Rout of HIV transmission	
10	Stage of HIV infection	
11	Number of CD4 lymphocytes /date	
12	Secondary diseases/date	
13	Concomitant diseases/date	
14	Antiretroviral treatment (when he/she started, how he/she tolerates, what medications he/she receives)	
15	Were there any interruptions in treatment? What are they related to?	
16	Other HIV patients in the family (who?)	
17	TB patients in the family (presence of MDR)	
18	The Mantoux test results (in childhood, now)	
19	Physiological anamnesis (contact with a patient who had tuberculosis, was registered at TB clinics or centers)	
20	Stay in penitentiary institutions (not been, less than a year, 1-3 years, more than 3 years)	

21	The last radiological scanning of the lungs (date)	
22	Chemoprophylaxis of tuberculosis (earlier and last)	
23	Who issued the anti-TB drugs	
24	The period of time for receiving anti-TB drugs (the dates of the beginning and end)	
25	At what time did you take anti-TB drugs?	
26	The regularity of receiving anti-TB drugs	
27	Did one of the relatives help to control the intake of drugs?	
28	Causes of irregular medication intake	
